# Economic Recovery in the Age of COVID-19

**DOI:** 10.1007/s10272-022-1019-8

**Published:** 2022-02-12

**Authors:** Carlos Cuerpo

**Affiliations:** Secretary General of the Treasury and International Financing, Tesoro Público, Paseo del Prado, 4, 28014 Madrid, Spain

**Keywords:** E62

## Abstract

Economic recovery post-COVID-19 will be structured around answering a number of fundamental questions. It is necessary to take stock of the extent of the recovery and of the transformation our economies have experienced in the last year and a half.

Economic recovery post-COVID-19 will be structured around answering a number of fundamental questions. It is necessary to take stock of the extent of the recovery and of the transformation our economies have experienced in the last year and a half. To move forward, an assessment of the response to the pandemic undertaken at the European and national level to achieve the recovery must be considered. Finally, there needs to be a discussion on common priorities for what lies ahead. This will naturally include fiscal rules, but should also focus on the global picture. There needs to be consistency in the wide range of interdependent policies that are part of our common agenda going forward.

## Recovery is getting traction

Although recent developments, i.e. rising COVID-19 infection rates and the emergence of a new variant, show that the pandemic is not yet behind us, the fact that we are able to talk about the future of the EU highlights how much things have improved, and although risks and uncertainty are still present, the overall balance of risks has become brighter. The recovery of our economies is outperforming initial forecasts and is more robust than anticipated only a few quarters back. This is mainly the result of the bold and coordinated economic policy response adopted at the national and European levels, which, together with high vaccination rates, has allowed us to lay a solid foundation for recovery.

We are fortunately witnessing a turning point in the economic situation in Europe, with clear signs of recovery. In Spain, together with high vaccination rates, growth has accelerated over the course of 2021, and the labour market is showing outstanding dynamism. We have experienced an unprecedented crisis. These past 23 long months have surely changed many things. The virus hit us when we had barely recovered from the previous financial crisis: It took Spain ten years to recover the pre-2008 GDP levels and 12 years to recover employment levels. In some instances, such as inequality, we had not recovered from the financial crisis when this new pandemic hit us.

**Carlos Cuerpo**, Tesoro and International Financing, Madrid, Spain.

This time is proving to be different in many respects. For example, it is worth taking a look at the response of the elasticity of employment and public revenues to GDP developments. Employment has recovered sharply, reaching its highest level since 2008 with around 20 million people employed and the number of workers under short-term work or furlough schemes declining rapidly. The pre-crisis levels of employment as well as other indicators such as unemployment and youth unemployment have already recovered. This is clearly much better than any analyst or institution would have anticipated only one year ago.

Reaching the current situation has only been possible thanks to the unprecedented innovative and coordinated response to the crisis that has been implemented both at the national and European level.

## An innovative and coordinated response to the crisis

First, the coordinated response at the national level included short-time work schemes, public guarantees and a wide variety of measures to support households and firms. This was partly possible thanks to the activation of the general escape clause within the Stability and Growth Pact and the shift towards a more qualitative surveillance in the EU.

Second, the decisive response of European institutions, including the ECB and the European Commission, proved once again, resilient in the midst of a crisis.

Third, common safety nets, including the temporary Support to mitigate Unemployment Risks in an Emergency (SURE), an embryo of a potential unemployment reinsurance scheme, have proven to be highly effective in supporting families and firms.

This time our collective response has been different and we should be proud of what has been achieved. We have provided a response that is not only different in size, but also in substance. It was a response consistent with our vision for more sustainable and inclusive economies that brings member states closer together rather than further apart. We have ensured a stable financial framework that has helped to prevent a global financial crisis and we have allowed national governments to make the right calls to minimise structural damage and protect a basis for the recovery. The bottom line is that common and coordinated solutions are clearly worthwhile. Now we must continue our support in order to prevent the economic recovery from derailing rather than wait and see if the rebound holds itself. As the saying goes, it is always better to be safe than sorry.

First, we should avoid cliff-edge effects, encompass the phasing in of economic activity with the targeting of support measures towards more needed sectors, making sure that the transition to recovery mode is smooth. As highlighted above, we have already seen the benefits of prevention through the emergency response to the crisis and we have successfully prevented scarring effects.

At the same time, we are already gradually shifting from an emergency mode to a recovery mode. This requires a transition from broad measures to more targeted actions to facilitate the reallocation of resources and at the same time protect the most affected and vulnerable parts of society.

In the short term, fiscal policy needs to be supportive until the recovery takes hold and uncertainty starts to recede. Uncertainty is still very high due to new waves of COVID-19 rapidly expanding in many countries. Additionally, supply-side disruptions, together with the increase in energy costs, are leading to short-term inflationary pressures. These developments show that we should be prudent and avoid complacency, remaining flexible in our policy response and support.

## Key aspects for the future of Europe

As we are emerging from the crisis, the challenge is to make sure it is not a short-run bounce back but a real transformative process that puts the EU on a sustainable and stronger growth path from the economic, environmental and social perspectives.

National and European agendas have been moving from an emergency mode to a recovery mode, focusing on structural policies that will be key in the medium and long term. This is an important reform momentum both for member states and for the EU. The coming months open a window of opportunity to shape the recovery and the economic model for the future that we should not miss. At the national level, this will be done with the implementation of the Recovery and Resilience Plans, and at the EU level, with an intensive discussion on key strategic fields among which the economic governance review stands out as a top priority in the European agenda.

The NextGenerationEU plan will be instrumental. At a national level, ambitious recovery plans have generated positive expectations among businesses, citizens and society in general. Now that the whole system is finally up and running, and that reforms and investments are accelerating, we expect to see a very substantial impact on the real economy in the short term but also in the long term, reinforcing potential output. The combination of investment and reforms is a very powerful one.

The Spanish plan is particularly ambitious, with €69.5 billion in grants, which will be complemented with a comparable volume of loans at a later stage. This is an unprecedented volume of funds, even compared with funds historically received through the EU budget. Therefore, the plan will play an essential role in Spain to address the significant investment needs going forward. In fact, investment dropped dramatically in the last crisis and had not recovered since. Public investment usually suffers from post-crisis consolidation efforts, hampering long-term growth potential. The plan will hopefully reverse this trend, providing a much needed boost to investment in Spain.

Beyond the volumes involved, the Spanish plan's ambition stems from the fact that structural reforms are placed on an equal footing with investments. We have already carried out an important reform effort over the past year and a half, and we have frontloaded the most relevant reforms of the plan to the first two years. This specific design will reinforce the effective implementation of investments.

Of course, the successful implementation of reforms and investments is not only important for Spain. Given the strong interlinkages between our economies, we should all be equally interested not only in deploying our respective national plans, but also in making sure that all plans are successfully approved and implemented, as they could foster beneficial spillovers across the EU. Because the more our European neighbours grow, the more Spain will grow and vice versa.

There is a qualitative element related to the focus of the plan and of NextGenerationEU: The twin transitions are clear priorities for the future of Europe, and thus, they imply investment needs that will have to be addressed in the coming decades.

On the green transition, we have to deliver on ambitious objectives under the European Green Deal, which provides a compass for our joint action. Besides the key contribution of the recovery plans, we will strive to make progress on other fronts, starting with the Commission's upcoming proposals under the Fit for 55 package. This is also a matter of intergenerational fairness. The consequences of not acting now are difficult to assess accurately, but the best and most recent evidence suggests that major disruptions could occur in the absence of bold preventive actions. Not doing enough now would mean increasing future environmental, social and economic risks – in other words, building up significant contingent liabilities that may threaten our future. For this, mobilising public and also private funds will be needed.

It is evident that the future of Europe is more digital. We should trust the enormous opportunities for citizens, businesses and public administrations that this process brings. There is a huge opportunity cost of not investing sufficiently today in the digital technologies that will determine the competitiveness of Europe tomorrow. We must embrace this historical opportunity and avoid that Europe falls further apart in the global scenario, where other international players are rapidly moving forward.

Overall, national recovery plans are instrumental to pivot from emergency to transformation mode. They must be the key lever that allows for an exit strategy that delivers while also encouraging a growth-friendly consolidation path that avoids cliff effects and accommodates the ambitious investment programmes and transformative reforms that the twin transitions require. In this context, the relaunch of the debate on the economic governance review is particularly timely.

### The urgency to reform has increased

The Communication on the EU economy after COVID-19 that was issued by the Commission in October 2021 confirms that the shortcomings that were evident before the pandemic have not disappeared, they have become even more relevant.

First, government debt levels have increased substantially since the outbreak of the pandemic. In many European countries, there are high levels of public debt, and the question we must answer is how the adjustment can be done more efficiently. Ensuring public debt sustainability is an issue that we will have to address by undertaking growth-friendly consolidation strategies.

Second, as mentioned above, in the coming decade we will need to undertake a bold agenda of investments in order to keep up with the green and digital transitions. The investment gap will be in the range of €650 billion per year according to the Commission's estimates. Since investment needs are shared and spillovers across member states can be significant, there is a clear case for closer European coordination under a common framework, to foster our global competitiveness and increase the well-being of future generations of Europeans, thereby ensuring intergenerational fairness. We need to prevent investment from becoming, once again, the first victim of our consolidation strategies.

### Avenues for reforming the fiscal rules

Discussions will intensify in the coming months with the aim of reaching a broad-based consensus in time for 2023. These discussions are likely to be complex. However, it is possible to push for an ambitious calendar of negotiations and have quality results particularly by acknowledging that there is already a good basis for consensus. For example, there is a shared need to reform the current framework to make it simpler, more realistic, credible and to improve enforcement. A consensus can be built based on three crucial ideas.

First, there is a need to define credible public debt reduction strategies. And in order to be credible, these strategies cannot jeopardise the recovery; they need to be growth-friendly. Long-term debt sustainability also depends on the ability to have long-term growth.

Second, we should address the investment gap by mobilising public and private investments in areas such as the green and digital transitions. This in an unprecedented situation where we are facing global challenges. We need to create a virtuous circle in which both financial and environmental liabilities are reduced. We need to find ways to avoid repeating past mistakes and strike a better balance between consolidation and investment needs.

Third, the commitment-based approach of the Recovery and Resilience Facility and its multiannual framework can be a useful source of inspiration for reforming the Stability and Growth Pact and the Macroeconomic Imbalances Procedure, promoting a virtuous circle of increased national ownership and improved enforcement that could pave the way for the design of multiannual fiscal frameworks. Finally, when addressing credible debt reduction strategies, a politically feasible and commitment-based approach is the best way forward in this regard.

This will not be the last crisis. There will inevitably be others in the future and therefore we need to be better prepared by strengthening the stabilisation role of national fiscal policies as well as by strengthening the architecture of the Economic and Monetary Union.

## Conclusion

The pandemic has required unprecedented solutions to our common problems. Once again, it is clear that more Europe brings better solutions to our citizens. We collectively have to ensure the recovery process is a success, increasing potential growth, creating good quality jobs, and aiming at a more sustainable and inclusive growth model. And we also need to design the right future-proof framework to finance the necessary public investments and implement the necessary reforms aligned with our shared priorities. In other words: We need to get it right with the recovery like we did with the response to the pandemic.
